# Myocardial T1 maps reflect histological findings in acute and chronic stages of myocarditis in a rat model

**DOI:** 10.1186/s12968-016-0241-6

**Published:** 2016-04-16

**Authors:** Sarah Jeuthe, Katharina Wassilew, Darach O h-Ici, Tiago Ferreira da Silva, Frédéric Münch, Felix Berger, Titus Kuehne, Burkert Pieske, Daniel R. Messroghli

**Affiliations:** Internal Medicine/Cardiology, Deutsches Herzzentrum Berlin, Augustenburger Platz 1, 13353 Berlin, Germany; Cardiovascular Pathology, Cardiothoracic and Vascular Surgery, Deutsches Herzzentrum Berlin, Berlin, Germany; Congenital Heart Disease and Pediatric Cardiology, Deutsches Herzzentrum Berlin, Berlin, Germany

**Keywords:** T1 mapping, Autoimmune myocarditis, Lewis rat, Dilated cardiomyopathy, Cardiovascular magnetic resonance

## Abstract

**Background:**

Cardiovascular magnetic resonance offers both diagnostic and prognostic information in myocarditis. Using an established animal model of myocarditis, the aim of this study was to measure myocardial T1 before the onset, in the acute and in the chronic phases of the disease and to compare its course with histological and immunohistochemistry findings.

**Methods:**

Male young Lewis rats were immunized with 0.25 mg porcine myocardial myosin into the rear footpads on day 0. Native and contrast-enhanced ECG-triggered cardiac MRI examinations were performed before immunization on day 0 and on days 14, 21 and 35. Left ventricular function, pre- and post- contrast T1 parameters and LGE images were assessed using Small animal look-locker inversion recovery (SALLI). For each of the indicated time points a minimum of 4 rats were randomly sacrificed for pathological investigations including conventional histology (HE and Sirius-Red staining) and immunohistochemistry (CD 68) investigations.

**Results:**

All immunized rats developed myocarditis (morbidity 100 %). Histologically we observed increased wall thickness with biventricular macrophage-rich mixed inflammatory infiltrates. All rats with a histologically severe myocarditis showed increased native T1 and decreased post-contrast T1 of the myocardium.

**Conclusions:**

The assessment of native T1 and post-contrast T1 allows accurate differentiation between healthy myocardium and myocardium with inflammation and also between the acute and chronic phases of the disease.

## Background

In myocarditis, inflammation within the myocardium can lead to myocyte necrosis and subsequent myocardial fibrosis. Myocarditis may be caused by many agents (viruses, bacteria, drugs, toxins, autoimmune) and presents with a wide range of symptoms from transient fatigue, acute chest pain to heart failure or sudden cardiac death [1–3]. In addition, a significant proportion of dilated cardiomyopathies may be caused by unrecognized myocarditis [4, 5]. Because of the heterogeneity of symptoms, the diagnosis of myocarditis is challenging [6, 7]. Endomyocardial biopsy (EMB) with (immune-) histology is the gold standard for the diagnosis of myocarditis. However, EMB is an invasive method with a low sensitivity (20–75 %) [8–10] due to sampling error (if the sample is taken from a non-involved area of the myocardium) [3, 11] and with significant risk (perforation, tamponade, in 0.1–0.5 % of the patients [12, 13]. Cardiovascular Magnetic Resonance (CMR) is increasingly used as a non-invasive diagnostic tool for patients with suspected myocarditis [7, 14–17]. CMR is the non-invasive reference standard for the quantification of ventricular volumes and function. The same CMR method used for the assessment of viability in coronary artery disease (Late Gadolinium Enhancement; LGE) can be used to visualize focal lesions in myocarditis [18]. However, conventional CMR methods aiming to assess diffuse, non-focal involvement of the myocardium suffer from large overlap between normal and disease states [19]. Thus, a non-invasive method that allows the detection of widespread inflammation and diffuse fibrosis is needed. Cardiac T1 mapping is a parametric CMR method allowing for characterizing the myocardium by directly quantifying the signal intensity. In contrast to conventional CMR methods, T1 mapping can detect not only focal changes but also global myocardial injury without the need for an extra-cardiac reference tissue [20–23]. Thus, T1 mapping is a non-invasive imaging method that may help in the diagnosis and follow-up of patients with myocarditis without the risks of invasive biopsy.

Experimental autoimmune myocarditis (EAM) is a well-known myocarditis model in rats, which mimics human myocarditis in the acute and chronic phases [24–26]. The histological findings in EAM are similar to human myocarditis, with fibrosis and myocyte necrosis involving mononuclear cell infiltration [27–29].

We aimed to study the changes in myocardial T1 in the course of myocarditis. Using EAM rats, we assessed the myocardium at baseline and then in the acute and chronic stages of myocarditis, and compared the results with histological and immune-histological findings.

## Methods

### Experimental procedures

Male Lewis rats (Janvier, Le Genest-St-Isle, France) with a bodyweight of 230–260 g were used in this study (*n* = 35). Rats were housed in a specific pathogen free (SPF) barrier under standardized conditions with controlled temperature and humidity and a defined circadian rhythm of 12 h each of daylight and darkness. They were kept in groups of four or five with free access to food and water. All procedures and experimental protocols were performed in accordance with the *Guide for the Care and Use of Laboratory Animals* published by the US National Institutes of Health and were approved by local authorities (Landesamt für Gesundheit und Soziales; G0276/11).

### Study design

We immunized the rats as previously described with a myosin dose of 0,25 mg to the rear footpads on day 0 [30]. Functional and morphological CMR was performed on days 0, 14, 21 and 35. At each of these time points, at least 4 rats were randomly sacrificed for histological analysis.

### Cardiac morphometric parameters

The bodyweight (BW) of rats was measured directly before performing CMR. Directly following CMR the rats were sacrificed, and the heart was removed and weighed to calculate the ratio of heart weight to body weight (HW/BW).

### CMR protocol and image analysis

All CMR studies were conducted on a 3 T clinical system (Achieva, Philips Healthcare, Best, The Netherlands) equipped with a dedicated coil for rats. Animals were positioned in the coil head first and supine with ECG electrodes placed on the rats’ feet. Anesthesia was maintained throughout the examination via inhalation of 1–2 % isoflurane. Using a long-axis set of cine images (2-chamber view and 4-chamber view) and a left ventricular (LV) cine short-axis stack (phases, 30; repetition time (TR), 15.8 ms; echo time (TE), 4.60 ms; flip angle, 15°; field of view, 80 × 64 mm; acquired voxel size, 0.4 × 0.4 × 1.5 mm; number of signal averages, 4; slices, 7; slice thickness 1.5 mm) the following parameters were assessed: LV ejection fraction, end-diastolic volume, end-systolic volume and cardiac output. After functional analysis, T1 parameters were measured before and after gadolinium contrast injection (gadobutrol [Gadovist]; Bayer-Schering AG, Berlin, Germany, 0.1 mmol/kg) at the same location in a midcavity short-axis using Small Animal Look Locker Inversion Recovery as described previously [22, 31], but with optimized parameters (SALLI; acquisition duration, 4000 ms; relaxation duration, 4000 ms; phases, 4; flip angle, 10°; field of view, 64 × 64 mm; acquired voxel size, 0.6 × 0.6 × 2.4 mm; TR, 7.6 ms; TE, 3.1 ms; number of signal averages, 4; total acquisition time, 7 min 20 s). SALLI also generates Late Gadolinium Enhancement (LGE) images during the same acquisition. Matlab was used for the reconstruction of the T1 maps from the SALLI data sets. The native (pre-contrast) and post-contrast T1 of the LV myocardium and blood pool were recorded from the region of interest (total left ventricle including the septum) in the SALLI T1 maps using Image J64 (Rasband, W.S., ImageJ, U. S. National Institutes of Health, Bethesda, Maryland, USA, http://imagej.nih.gov/ij/, 1997–2014). LGE images were evaluated using a relative score system: 0-no visible Late Gadolinium Enhancement; 1- pericardial location of Late Gadolinium Enhancement; 2-epicardial location of Late Gadolinium Enhancement; 3-midwall location of Late Gadolinium Enhancement). LV function parameters were analyzed in a CMR analysis software package (CMR42 version 3.4.1; Circle Cardiovascular Imaging Inc, Calgary, Alberta, Canada).

### Serological analysis

Blood samples were taken on day 0, 14, 21 or 35 directly before the rats were sacrificed. Troponin T, a sensitive marker for cardiomyocyte damage [32], was measured by an external laboratory (synlab.vet GmbH, Turmstraße 21, 10559 Berlin, Germany).

### Histological and immunohistochemical analysis

After the final CMR scan, the heart was explanted and fixed in warm 4 % buffered formalin. It was then embedded in paraffin and stored at 21 °C. The paraffin-embedded sections of the mid-ventricle were stained with hematoxylin and eosin (HE) for general analysis and with a collagen stain (Sirius-Red) to detect fibrosis. The collagen volume fraction (CVF) was calculated as the fraction of the pink-colored collagen of the total area [33]. Immunohistochemistry was performed on the formalin-fixed, paraffin-embedded cardiac tissue sections to study the components, quantity and distribution of the mononuclear inflammatory infiltrate with a pan-macrophage marker (Mouse Monoclonal antibody to CD68, GeneTex Inc.; secondary antibody from Bond-Max-Kit, Post Primary AL rabbit-anti-mouse-IgG; Polymer Ap anti-rabbit-poly-Ap-IgG). The severity of myocarditis was analyzed on conventional histology, including features as percent of involved cardiac tissue mass by necrosis, edematous widening of the interstitium and within presence of acute inflammatory cells as neutrophils. CD68 staining (Keyence BZ 9000 + analysis software). Additionally, we calculated the percentage infiltration as the fraction of the stained macrophages of the total area (Keyence BZ 9000 + analysis software).

### Statistical analysis

We used GraphPad Prism 5 software (San Diego, CA, USA) for statistical analysis. A Mann Whitney test was used to compare cardiac morphometric parameters, blood analysis and histology within group 0 days and groups 14, 21 or 35 days. We used a Wilcoxon matched-pairs signed rank test for the MRI parameters (T1 pre –and post contrast, SV and EF). Results were considered to be significant at *P* < 0.05.

## Results

### Cardiac morphometric parameters

Heart weight/body weight ratio was significantly higher in animals with EAM compared to healthy animals (see Table 1). This increase reached a peak at 14 days and gradually declined, but did not return to control values by 35 days.

**Table 1:** Course of Troponin T and variation of heart weight to body weight ratio in EAM. Table shows serological changes in Troponin T levels and changes in ratio of heart weight to body weight (HW/BW) after 0, 14, 21 and 35 days after immunization. Results are presented as the mean ± SEM. *p < 0.05, **p < 0.01 vs. 0d

Timepoint after immunization	Troponin T μg/L	HW/BW Ratio
0 days	1,06 ± 0,58	0,36 ± 0,06
14 days	6,35 ± 1,42**	0,63 ± 0,2**
21 days	4,13 ± 1,35**	0,6 ± 0,08*
35 days	0,31 ± 0,48**	0,43 ± 0,15**

### Blood analysis

Animals with EAM had significantly higher levels of troponin T on day 14 and 21 after immunization compared to animals before immunization on day 0 with healthy myocardium (see Table 1).

### CMR findings

**Fig. 1:**
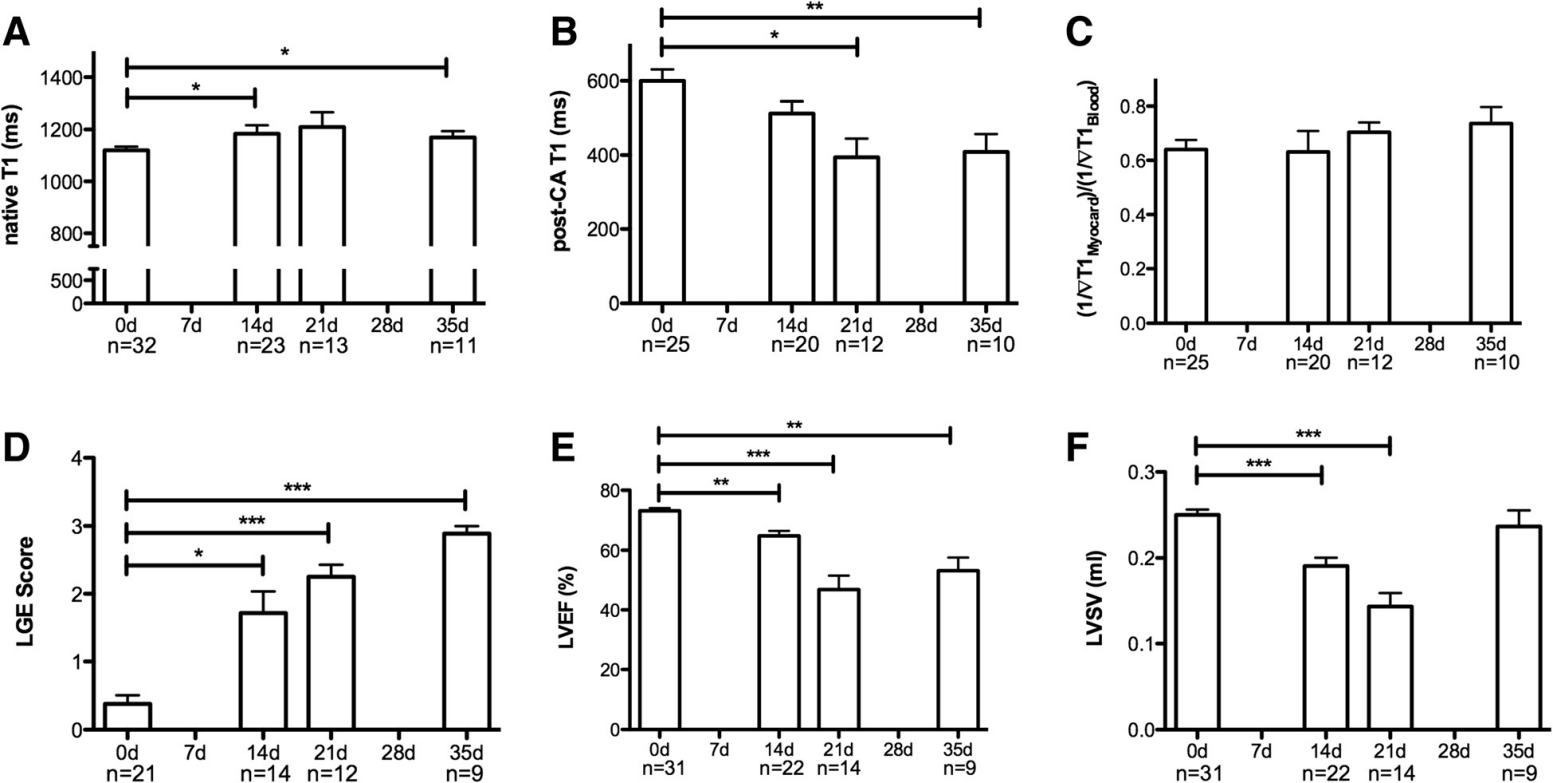
Functional and morphological CMR results in EAM. Bar graphs show changes in native T1 (**a**), post-contrast T1 (**b**), partition coefficient (**c**), LGE Score (**d**), LVEF (**e**) and LVSV (**f**) between rats before immunization (day 0), 14, 21 and 35 days after immunization. Results are presented as the mean ± SEM. *p < 0.05, **p < 0.01, ***p < 0.001 vs. 0d

LV functional parameters (EF and SV) show significant differences between rats before and after immunization. EF and SV decreased in the course of the disease with the lowest values occurring on day 21. By day 35, values had begun to recover, but remained below the baseline myocardial function. Native T1 was significantly increased at day 14 compared to day 0. Native T1 was also increased on day 21 and 35, but these values were not statistically different from those at day 0. Post-contrast T1 was significantly decreased on days 14, 21 and 35 when compared to day 0. LGE was found predominantly in the outer third of the myocardium 14, 21 and 35 days after immunization (see Fig. 1).

### Histological and immunohistochemical analysis

**Fig. 2:**
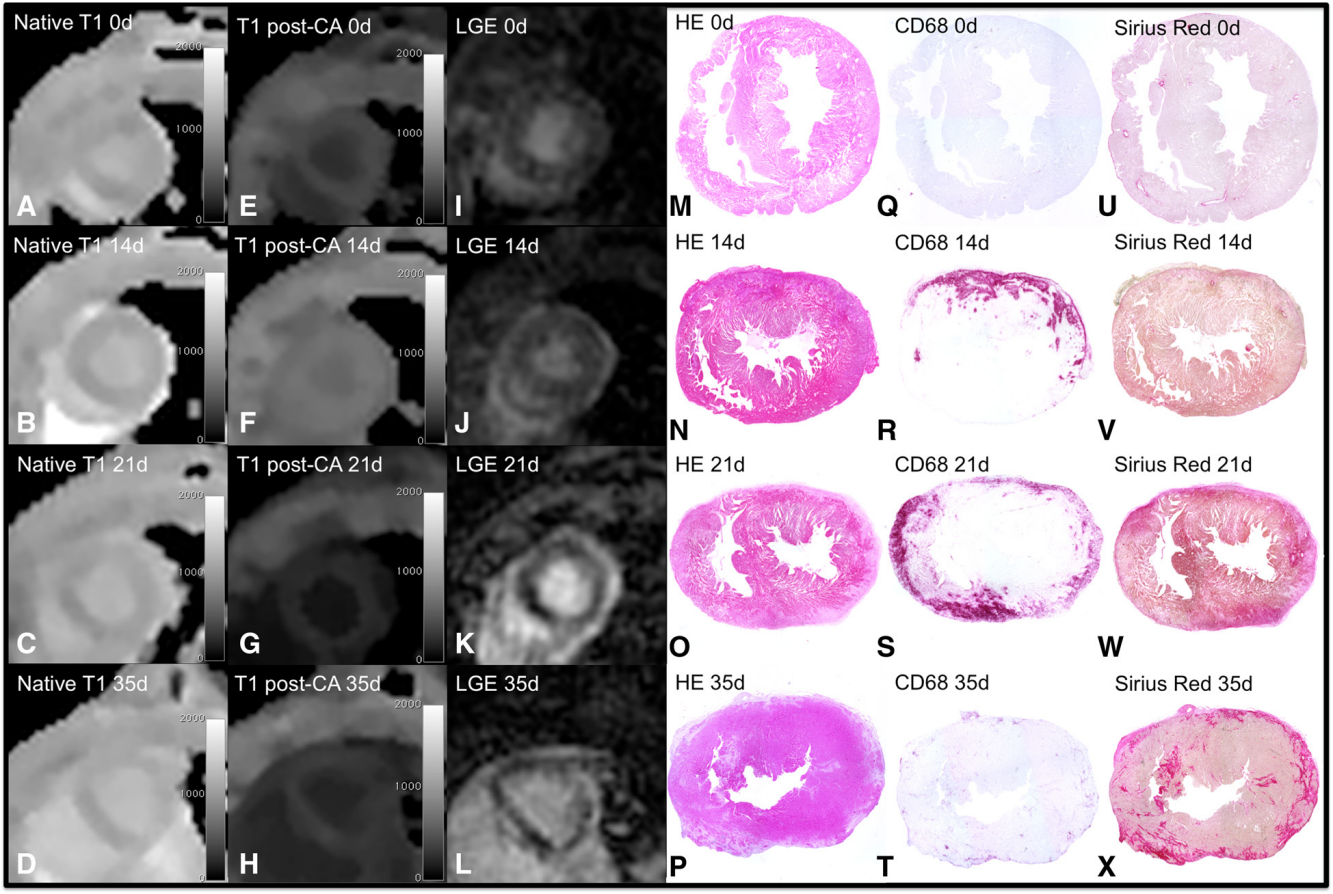
Comparison of CMR and histological findings in different stages of EAM. Pictures show histological (CD68-staining **q**–**t**, HE-staining **m**–**p**, Sirius Red-staining **u**–**x**), T1 (native T1 short axes images **a**–**d**, post-contrast T1 short axes images **e**–**h**) and LGE (LGE short axes images **i**–**l**) changes in rats before immunization (day 0), 14, 21 and 35 days after immunization

**Fig. 3:**
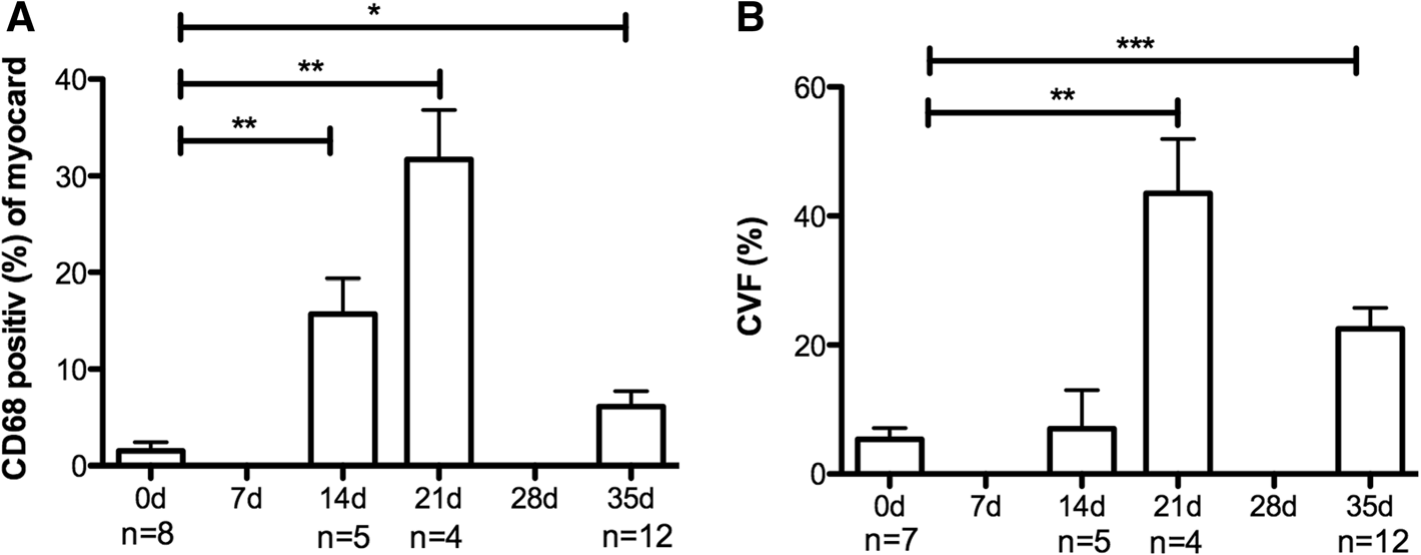
Histological results in different stages of EAM. Bar graphs show histological changes before immunization (day 0), 14, 21 and 35 days after immunization. **a** CD68-staining, **b** Sirius Red-staining. Results are presented as the mean ± SEM. *p < 0.05, **p < 0.01, ***p < 0.001 vs. 0d

All hearts from animals before immunization on day 0 presented with healthy myocardium. The hearts of the rats 14 and 21 days after immunization typically demonstrated a marked inflammatory infiltrate in both ventricles. 35 days after immunization, this infiltrate was less marked (see Fig. 3, CD68). CVF was significantly increased to day 0 in rats with EAM (21 and 35 days; see Figs. 2 and 3, Sirius-Red). Pericardial effusion was found in 4 of 5 animals 14 days after immunization, 3 of 4 animals 21 days after immunization and 3 of 12 animals 35 days after immunization. We did not note any pericardial effusion before immunization (day 0).

## Discussion

In this study we found that T1 mapping could detect characteristic changes of myocardial T1 throughout the course of EAM, reflecting progress and regression of inflammatory infiltrates and fibrosis. We measured T1 times on day 14, 21 and 35 after immunization and compared them to histology and immunohistochemistry results. Native T1 and post-contrast T1 reliably detected EAM. Changes in myocardial function and myocardial composition could be detected at each time phase.

In patients with acute myocarditis, first clinical studies [34–37] demonstrated that T1 mapping has a higher sensitivity as compared with T2W-CMR and LGE techniques. In these studies however, no histological samples were obtained. Thus, while these studies demonstrated the potential of native T1 mapping for diagnostic use in acute myocarditis, the underlying mechanisms of the T1 behavior and its further course over time remained unclear. In our study, the use of a well-established preclinical model of EAM allowed us to correlate T1 changes to underlying pathology at different stages of the disease.

### T1 mapping detects inflammation: comparison of histology findings and CMR analysis

From our findings and the results of previous studies, it could be said that day 14 reflects the onset of the disease, day 21 the peak acute inflammatory phase and day 35 the chronic state of the disease. On day 14 after immunization macrophage infiltrates increase to reach a peak on day 21. This acute inflammation is nearly complete by day 35, where macrophage infiltration had decreased to near baseline. Histologically, myocardial fibrosis peaked at day 21, and had decreased by day 35, but remained still higher than baseline values from day 0 (see Figs. 2 and 3, CD68 and SiriusRed).

Our MRI findings were matched by histology. Native and post-contrast T1 mirror the histological findings throughout the course of the disease. Native T1 increases to reach a peak at day 21, thereafter decreasing by day 35. Post-contrast T1 followed the opposite course, reaching a nadir at day 21 before starting to increase at day 35. The course of myocardial T1 values corresponds to the course of the inflammatory infiltrates throughout the disease. It is already known that post-contrast T1 is reduced in the presence of fibrosis [38]. We suspect that post-contrast T1 is also reduced in the presence of inflammatory infiltrates but its shortening only reaches its full extent with the advent of myocardial fibrosis. 14 days after immunization we detected histologically a slight increase in fibrosis and a massive increase of inflammatory infiltrates, but a significant decrease in post-contrast T1 and a significant increase in native T1 compared to healthy myocardium. We conclude that the increase in native T1 is predominantly driven by cellular infiltration and the corresponding edema, while post-contrast T1 shortening mainly reflects collagen accumulation i.e. myocardial fibrosis.

### Study limitations

The study shows differences in the course of native T1 and myocardial partition coefficient during the course of EAM. An assessment of ECV was not possible because hematocrit was not available for all animals. ECV is regarded as the parameter of choice for the assessment of contrast distribution in human studies because of its robustness against confounders such as differences in contrast dose and timing, body weight, and renal function, all of which are less critical in preclinical studies.

To avoid longer scan times with a risk of higher mortality of the animals, T1 mapping was not assessed across the whole heart but only in a single midcavity short-axis slice. Inflammatory changes could be observed in all animals with this approach.

This study is based on an autoimmune myocarditis model, while in humans myocarditis is believed to be caused by viral infection. However, this viral infection essentially acts as a trigger for the onset of the disease, while the injury to the myocardium (by virus strains that otherwise cause minor infections) is created to a large extent by the abnormal immune response of the patient [39, 40]. EAM in rats leads to myocardial damage resembling that of (severe) human myocarditis, which is why we do not think that the different trigger mechanism limit transferability of the current findings to humans.

## Conclusion

In conclusion, T1 mapping permitted noninvasive diagnosis of myocardial inflammation, assessment of inflammatory severity and also the monitoring of the course of the disease in a preclinical model of myocarditis.

## Abbreviations

BW: body weight
CMR: Cardiovascular Magnetic Resonance
CVF: Collagen volume fraction
EAM: experimental autoimmune myocarditis
ECV: Extracellular Volume Fraction
EF: ejection fraction
HE: hematoxylin and eosin
HW: heart weight
LGE: Late Gadolinium Enhancement
LV: Left ventricular
SALLI: Small Animal Lock-Locker Inversion Recovery
SPF: specific pathogen free
SV: stroke volume
TE: echo time
TR: repetition time
